# Right External Iliac Artery Resection and Reconstruction Because of Cecum Cancer Invasion: A Case Report

**DOI:** 10.7759/cureus.84916

**Published:** 2025-05-27

**Authors:** Tomohiro Nakajima, Yutaka Iba, Masayuki Ishii, Maho Toyota, Koichi Okuya

**Affiliations:** 1 Department of Cardiovascular Surgery, Sapporo Medical University, Sapporo, JPN; 2 Department of Surgery, Division of Gastroenterological Surgery, Sapporo Medical University, Sapporo, JPN

**Keywords:** arterial invasion, cecum cancer, combined resection, prosthetic vascular reconstruction, right external iliac artery

## Abstract

This report describes the case of a 69-year-old female patient. At the age of 68, she was diagnosed with cecum cancer, and infiltration of the right external iliac artery was detected. Owing to arterial infiltration, surgical resection was considered inappropriate, and a future risk of intestinal obstruction in the cecum region was anticipated. Therefore, a laparoscopic ileum-transverse colon bypass was performed. Subsequently, chemotherapy with the FOLFOXIRI (folinic acid, fluorouracil, oxaliplatin and irinotecan) regimen plus bevacizumab was administered for five months. Contrast-enhanced computed tomography revealed reduced infiltration of the right external iliac artery, prompting plans for ileocecal resection with combined resection of the right external iliac artery and iliopsoas muscle. After resection of the intestine, preserving the bypass site and tissue mobilization except the right external iliac artery infiltration site, systemic heparinization was performed. An 8 mm artificial vessel was then interposed to reconstruct the right external iliac artery using an end-to-end anastomosis technique. Lower extremity blood flow was unremarkable, and the postoperative course was uneventful. The patient was discharged on postoperative day 10 without complications.

## Introduction

Locally advanced colorectal cancer invading the external iliac artery is a challenging clinical situation. Traditionally, tumor involvement of the lateral pelvic sidewall, particularly with the encasement of major neurovascular structures, has been considered a contraindication for surgical resection because of the risk of catastrophic hemorrhage and poor oncological outcomes [1].

However, recent advancements in surgical techniques have demonstrated that en bloc resection of the iliac vascular system during pelvic exenteration can be safely performed with acceptable morbidity and improved R0 resection rates [2]. Radical resection of the involved external or common iliac arteries, often requiring vascular reconstruction with synthetic or autologous grafts, has been associated with long-term survival outcomes comparable to those achieved in patients with more centrally located tumors [3].

Furthermore, the shift from vessel-sparing approaches to radical en bloc vascular excision has significantly increased the likelihood of achieving R0 resection, particularly in patients with locally recurrent rectal cancer [4].

Here, we report a rare case of colorectal cancer invading the external iliac artery that was successfully managed with en bloc resection and vascular reconstruction, achieving curative (R0) resection.

## Case presentation

**Figure 1:**
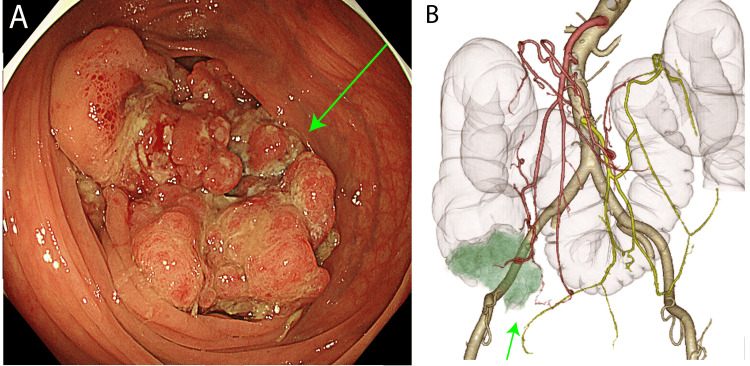
(A) Lower gastrointestinal endoscopy showing a 40 mm tumor present circumferentially in the cecum and was classified as type 2 (green arrow); (B) 3D-reconstructed CTA of the abdomen and pelvic blood vessels showing infiltration of cecum cancer into the right external iliac artery (green arrow). 3D: three-dimensional; CTA: computed tomographic angiography

The patient was a 69-year-old woman. One year earlier, at age 68, she experienced abdominal pain and diarrhea and was referred to our hospital from a nearby clinic for further examination at her request. During coloscopy, a 40 mm tumor was circumferentially identified in the cecum. Macroscopically, it was classified as type 2, and histological examination indicated a moderately differentiated adenocarcinoma with *BRAF* mutation. Further computed tomography (CT) imaging revealed cecal cancer with infiltration of the right external iliac artery (Figure 1).

**Figure 2:**
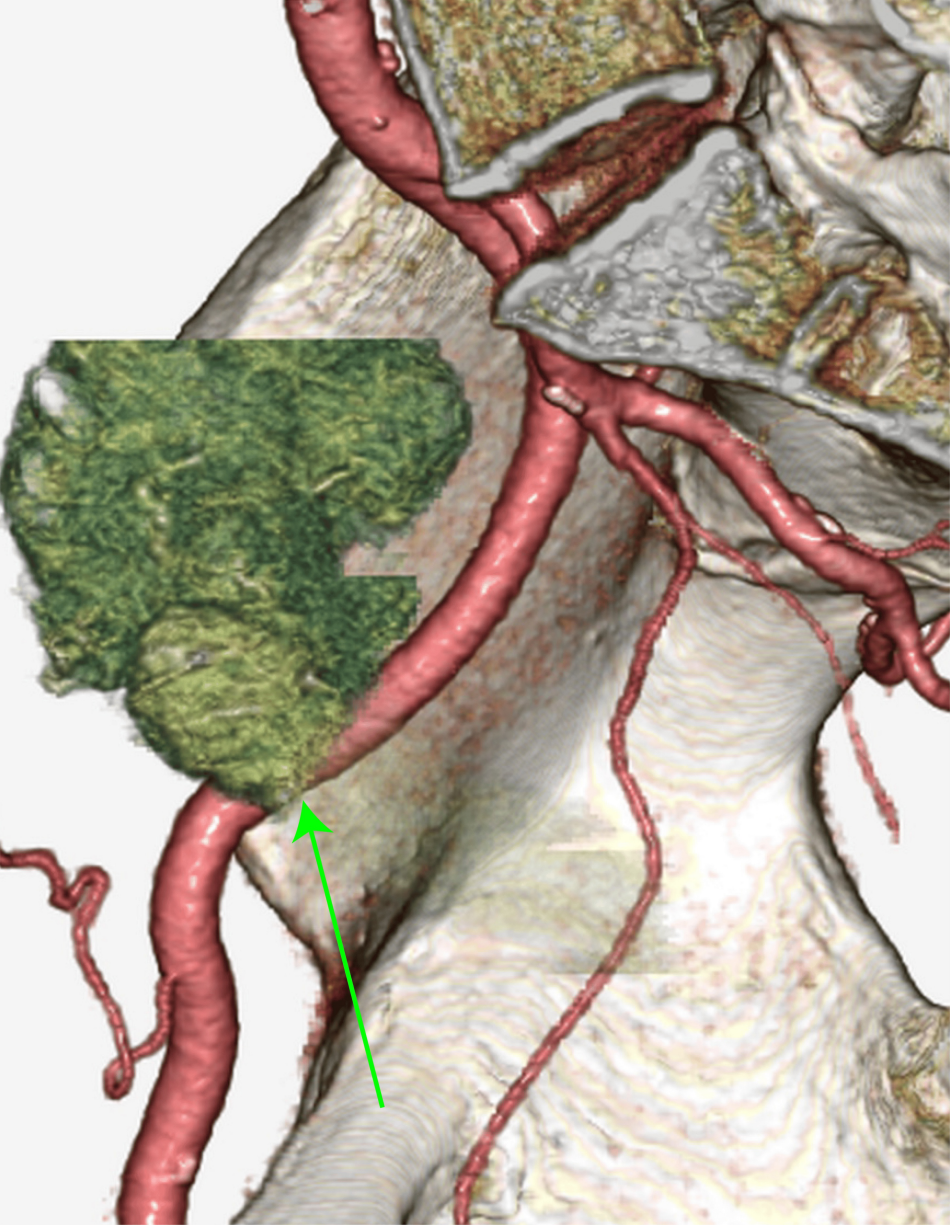
CT image (volume rendered) after chemotherapy shows that the extent of tumor infiltration into the right external iliac artery has decreased (green arrow).

Owing to arterial and iliopsoas muscle infiltration, surgical resection was considered inappropriate, and obstruction of the cecum was anticipated. After explaining the situation to the patient, an ileocecal bypass was performed to prevent future intestinal obstruction. Subsequently, chemotherapy with the FOLFOXIRI (folinic acid, fluorouracil, oxaliplatin and irinotecan) regimen plus bevacizumab was administered for five months. A contrast-enhanced CT scan was performed to evaluate the response to chemotherapy, which revealed a reduction in the infiltration of the right external iliac artery (Figure 2). 

Although radical resection was previously considered impossible because of the infiltration of the external iliac artery, resection of the cecal cancer along with the right external iliac artery was planned, and radical resection was performed. A consultation was conducted in our department, and it was determined that resection and reconstruction of the external iliac artery were feasible. Two reconstruction methods were considered for the external iliac arteries. The first option involves direct reconstruction using an artificial vessel within the abdominal cavity. This method is anatomically reconstructive but carries a contamination risk if the intestine opens within the abdominal cavity. The second option involves performing a femoral artery crossover bypass to maintain blood flow to the right lower limb and closing the distal end of the right external iliac artery. Although this method is non-anatomical, it has the advantage of preventing the contamination of artificial vessels if the intestine opens in the abdominal cavity. After discussing the two methods, we decided to proceed with the first option for this case, as the possibility of the intestine opening was considered low because of the previous surgery, which included an ileum-transverse colon bypass.

**Figure 3:**
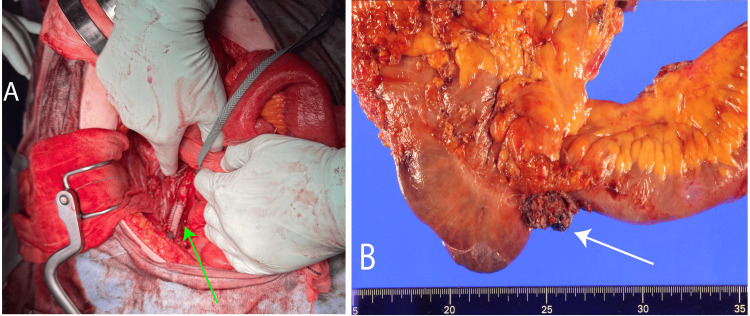
Intraoperative Images (A) The right external iliac artery is replaced with a synthetic graft (green arrow); (B) Pathological tissue specimen photograph showing the right external iliac artery infiltrated by the tumor (white arrow).

Surgery was performed under general anesthesia. The adhesions were dissected, and the ileum and transverse colon were resected with a stapler, preserving the bypass site and tissue mobilization excluding the region of infiltration into the right external iliac artery. The entire body was heparinized, and the right external iliac artery was occluded. The external iliac artery was transected with a 2 cm margin from the infiltrated area, and the tumor was resected en bloc. Subsequently, an 8-mm Gelweave® (Terumo Aortic, Inchinnan, Renfrew, United Kindom) was interposed, and the central and peripheral sides were anastomosed end-to-end using 5-0 Prolene (Ethicon Inc., Raritan, New Jersey, United States) sutures (Figure 3).

The abdominal wall was closed by the surgical team, and the procedure was completed. The right dorsal pedal artery and posterior tibial artery were palpable. Lower extremity blood flow was normal, and the postoperative course was uneventful. The patient was discharged on postoperative day 10. Two months postoperatively, the patient visited the outpatient clinic with no complications and continued to receive postoperative chemotherapy.

## Discussion

Arterial invasion by colorectal cancer, particularly involving the external iliac artery, is a technically challenging clinical situation [5]. Historically, such cases were deemed inoperable because of the high risk of catastrophic hemorrhage, graft infection, and poor oncologic outcomes. However, with advances in surgical techniques and perioperative management, combined resection and vascular reconstruction have become feasible options for achieving curative (R0) resection in selected patients [6].

In the present case, preoperative chemotherapy with the FOLFOXIRI regimen plus bevacizumab contributed to tumor downstaging and reduction of external iliac artery and iliopsoas muscle invasion, ultimately allowing for reconsideration of surgical resection. Preoperative chemotherapy has been shown to improve the resectability and long-term survival of patients with locally advanced colorectal cancer [7].

During surgical planning, two options for vascular reconstruction were carefully evaluated. The first option was anatomical reconstruction using a prosthetic graft placed intraperitoneally to bridge the resected external iliac arteries. This method provides greater physiological restoration of blood flow. However, it carries a significant risk of graft contamination if intra-abdominal contamination or bowel injury occurs during the procedure.

The second option was extra-anatomical reconstruction, specifically a femorofemoral crossover bypass, to maintain perfusion of the right lower extremity while closing the proximal stump of the right external iliac artery. Although this approach reduces the risk of prosthetic graft infection by keeping the graft away from a potentially contaminated intra-abdominal environment, it is non-anatomical, and long-term graft patency rates may be inferior to those of direct anatomical reconstruction [8]. Enari et al. reported an axillofemoral bypass for locally advanced sigmoid colon cancer invading the common iliac artery [9]. There is also one report of resection of a tumor infiltrating the iliac artery [10].

After a multidisciplinary discussion, we decided to proceed with the first option, anatomical reconstruction using an 8 mm prosthetic graft, based on the following considerations: The patient had previously undergone ileum-transverse colon bypass surgery without complications, the intra-abdominal field was expected to be clean, and the risk of intestinal injury could be minimized by performing bowel resection while preserving the bypass portion. Furthermore, the anatomical route was preferable to maximize long-term graft patency and physiological restoration of arterial flow.

Studies have reported that prosthetic grafts can be safely used even in potentially contaminated fields if strict infection control measures are implemented, including systemic antibiotics and careful handling during surgery [3]. In the present case, no graft-related infections or vascular complications occurred, and the postoperative course was uneventful.

Our experience highlights the importance of individualized surgical planning, taking into account not only anatomical factors, but also the risk of contamination and long-term vascular outcomes. Furthermore, the successful management of such cases emphasizes the critical role of a multidisciplinary team involving vascular surgeons, oncologic surgeons, and anesthesiologists. This case reinforces the growing body of evidence that aggressive surgical resection, including vascular resection and reconstruction, can be a curative option with acceptable morbidity and favorable oncological outcomes, even in patients with arterial invasion owing to colorectal malignancy.

## Conclusions

This report was about a case of colorectal cancer that infiltrated the right external iliac artery and iliopsoas muscle. Palliative intestinal bypass was performed to prevent obstruction, followed by chemotherapy. The FOLFOXIRI regimen chemotherapy was effective, and a reduction in the extent of infiltration into the external iliac artery was observed, allowing progression to curative surgery. The tumor was resected along with a portion of the external iliac artery, and the external iliac artery at that site was replaced with an artificial vessel.
